# Spontaneous Hepatic Hemorrhage Causing Acute Liver Failure Requiring Liver Transplant

**DOI:** 10.14309/crj.0000000000001268

**Published:** 2024-02-02

**Authors:** Krunal Shukla, Rahul Chaudhari, Amon Asgharpour

**Affiliations:** 1 Virginia Commonwealth School of Medicine, Richmond, VA; 2 Division of Gastroenterology, Hepatology and Nutrition, Department of Internal Medicine, Virginia Commonwealth University, Richmond, VA

**Keywords:** spontaneous hepatic hemorrhage, acute liver failure

## Abstract

Spontaneous hepatic hemorrhage (SHH) is a very rare but life-threatening entity that results from a breach in the hepatic parenchyma without any external cause, the most common being hepatocellular carcinoma and hepatic adenoma. We present a case of SHH without any underlying tumor or injury. The cause in our patient remained unclear, but we hypothesize that the patient's SHH was most likely coagulopathy-related.

## INTRODUCTION

Spontaneous hepatic hemorrhage (SHH) and liver rupture is an extremely uncommon surgical emergency that commonly results from benign or malignant liver tumors such as hepatocellular carcinoma (HCC), hepatic adenoma, focal nodular hyperplasia, hemangiomas, and metastases; hemolysis, elevated liver enzymes and low platelets (HELLP) syndrome; amyloidosis; and other causes.^1–3^ We present a case of a spontaneous liver rupture in a patient with no clear explanation for it.

## CASE REPORT

**Figure 1.**
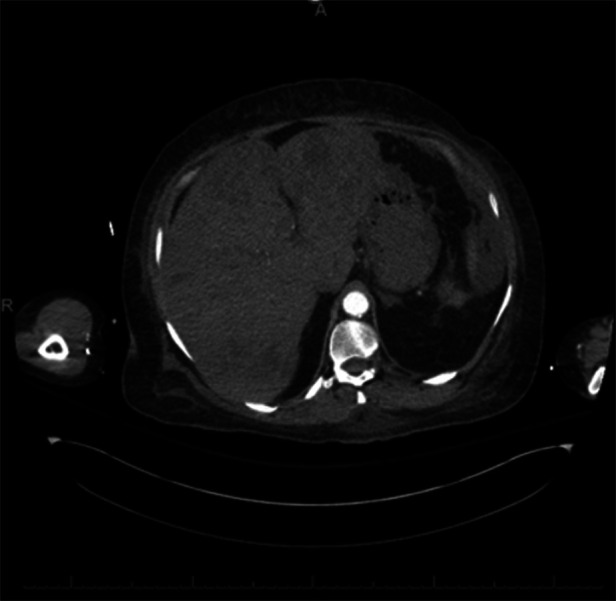
Abdominal computed tomography at time of admission showing cirrhotic liver morphology without any signs of laceration, hematoma, or malignancy.

A 59-year-old African American woman with a medical history of chronic kidney disease from membranoproliferative glomerulonephritis (MPGN), type 2 diabetes mellitus, hypertension, obesity, carpal tunnel syndrome, and anxiety was transferred to our tertiary care liver transplant center for worsening acute liver injury. The patient initially presented with a 3-day history of right flank pain. At admission, she was afebrile with a blood pressure of 175/88 and a heart rate of 117 bpm. Pertinent admission laboratory test results were notable for anemia, thrombocytopenia, and significantly elevated liver enzymes (Table 1). Of note, she was recently started on mycophenolate mofetil and prednisone by her nephrologist for the treatment of her MPGN about 2 months back. This was subsequently stopped once her liver enzymes started rising. After her admission, an initial computed tomography (CT) scan of the abdomen/pelvis and abdominal ultrasound indicated cirrhotic liver morphology with no evidence of active bleeding (Figure 1). Acute liver injury eventually progressed to acute liver failure complicated by acute anemia, thrombocytopenia, and systemic inflammatory response syndrome secondary to Gram-positive cocci bacteremia, requiring transfer to the intensive care unit.

**Table 1.** Initial admission laboratory test results showing elevated liver enzymes, indicative of acute liver injury

Laboratory test results	Value	Units
WBC	12.3	10^9^/L
Hemoglobin	7.5	g/dL
Platelet	47	10^9^/L
PT	17.3	s
INR	2.5	
Na	139	mmol/L
K	3.6	mmol/L
Creatinine	1.79	mmol/L
Protein	4.1	g/dL
Albumin	2.2	g/dL
Bilirubin	2.2	mg/dL
ALP	563	IU/L
ALT	3,625	IU/L
AST	2,598	IU/L

ALP, alkaline phosphatase; ALT, alanine transaminase; AST, aspartate aminotransferase; INR, International Normalized Ratio; PT, prothrombin time; WBC, white blood cell.

The initial workup for her liver injury remained inconclusive, including negative antinuclear antibody, anti-smooth muscle antibody, anti-liver kidney microsomal, anti-mitochondrial antibodies, normal alpha-1-antitrypsin, normal ceruloplasmin, negative viral serologies (hepatitis A/B/C, cytomegalovirus, Epstein-Barr virus, and herpes simplex virus), and normal iron studies. Liver biopsy at this point was deemed high risk because of the low platelet count and the high risk of hepatic hemorrhage. The underlying cause of acute liver failure was believed to be autoimmune hepatitis vs drug-induced liver injury, given a history of autoimmune disease and recent exposure to the mycophenolate mofetil. A decision to trial steroids was made, and liver enzymes started improving gradually.

**Figure 2.**
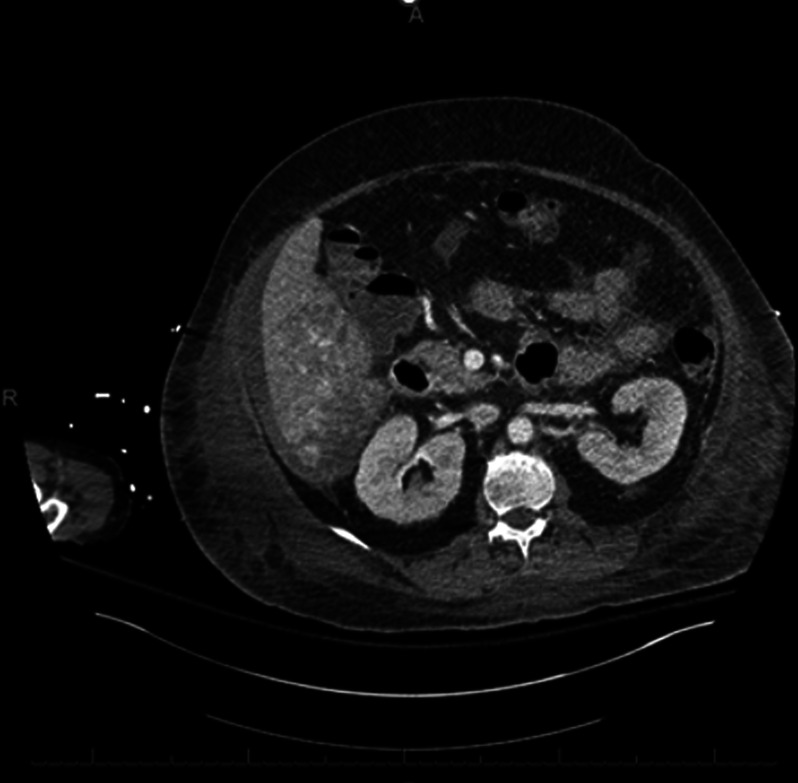
Abdominal computed tomography (3 days after admission) showing linear area of hypoattenuation within the inferior aspect of the right hepatic lobe extending to the periphery correlating to an appearance seen with laceration.

However, within 2 days of intensive care unit admission, the patient experienced altered mental status and clinically deteriorated. During this time, the patient's hemoglobin dropped from 9.1 to 5.1 without overt bleeding. A stat CT of the abdomen and pelvis with contrast was performed, which indicated right hepatic lobe laceration resulting in intraperitoneal hematoma with active extravasation in the absence of traumatic injury (Figure 2). A massive transfusion protocol, vasopressor support, and interventional radiology embolization eventually helped stabilize the patient. Her liver enzymes continued to get worse rapidly after this requiring an emergency liver transplant. The explanted liver showed extensive hemorrhage and necrosis, making it difficult to characterize the diagnosis histopathologically.

## DISCUSSION

SHH is a rare condition historically poorly recognized and infrequently diagnosed in patients presenting with shock. It contributes 1% of total admissions to specialist liver units. It is essential to identify it early and pursue appropriate interventions in a timely manner for this life-threatening condition.^4–6^

The etiology can vary widely, with HCC being the most common cause, with 10% of patients with HCC presenting with SHH. Patients with benign hepatic lesions such as hepatic adenoma, hemangioma, focal nodular hyperplasia, nodular regenerative hyperplasia, biliary cystadenoma, and angiomyolipoma could also present with SHH. Gynecological causes such as HELLP syndrome and fatty liver of pregnancy contribute to some of the cases reported in the literature. Other rare causes include peliosis hepatis, amyloidosis, systemic lupus erythematosus, and polyarteritis nodosa.^7,8^

The pathogenesis for SHH remains unclear and is believed to be multifactorial. In connective tissue diseases, poorly supported and weak liver parenchyma, when subject to trivial injury or physiological events such as changes in systolic blood pressure, results in SHH. In tumors and tumor-like conditions, 4 factors likely contribute to SHH: (i) disruption of a dependent artery/vein, traumatic injury of a superficial tumor from minor blunt abdominal trauma or from respiratory movements; (ii) splitting of overlying or adjacent hepatic parenchyma, particularly in cirrhosis, from pressure from tumor growth; (iii) fast-growing tumors with central necrosis may develop an expanding central hematoma and SHH because of compromise of the internal neovasculature; and (iv) coagulopathy. The incidence of SHH is not increased in patients with end-stage liver failure and refractory coagulopathy.^8^

The diagnosis depends on a high index of susceptibility because most of these patients present with nonspecific symptoms such as abdominal pain, nausea, and vomiting. SHH is not a common differential for these symptoms. Less than 10 percent of patients present with fever, jaundice, hematemesis, or chest pain. CT scan with contrast remains the gold standard test for diagnosis. Initial workup should include tumor markers such as carbohydrate antigen 19-9, carcinoembryonic antigen, and alpha-fetoprotein, along with other routine laboratory work.^7^

Moreover, without any underlying tumor or injury, our patient experienced significant right hepatic lobe laceration and intraperitoneal hematoma with active extravasation. We hypothesize that the patient's spontaneous bleeding was most likely coagulopathy-related. However, abdominal viscera are less commonly the sites of hemorrhage.^9^ For her acute liver failure, we considered all possible causes, but after extensive workup, autoimmune hepatitis (because of other autoimmune-related diseases) and drug-induced liver injury from mycophenolate (as liver injury started after starting mycophenolate for MPGN) seemed to be the likely causes.

Last, the management and prognosis of SHH are highly dependent on the extent and hemodynamic stability of patients. For relatively stable patients, nonoperative management with massive transfusion and/or transarterial selective embolization of affected vasculature has resulted in positive outcomes. For patients who fail to stabilize with nonoperative management or are hemodynamically unstable, urgent surgical intervention involving temporary tamponade of the liver using packs and portal triad occlusion through the Pringle maneuver should be pursued.^10,11^ A liver transplant might be needed for significant liver injury after such stabilization as in our case.

In conclusion, SHH is very uncommon. We present a case that is the first to the best of our knowledge that did not involve any malignancy or comorbidities typically associated with it in the setting of acute liver failure. We also have baseline imaging that helps us to further elucidate the timeline of the hemorrhage. It is essential to identify it early and pursue appropriate interventions for this life-threatening complication.

## DISCLOSURES

Author contributions: K. Shukla, R. Chaudhari, and A. Asgharpour wrote, revised, and approved the final manuscript. R. Chaudhari is the article guarantor.

Financial disclosure: None to report.

Informed consent was obtained for this case report.
